# “There just isn't any other option—so we just have to put up with it”: mental health in women's cycling and the necessity of structural change

**DOI:** 10.3389/fspor.2023.1270957

**Published:** 2023-11-16

**Authors:** Jill Colangelo, Alexander Smith, Anna Buadze, Michael Liebrenz

**Affiliations:** ^1^ Department of Forensic Psychiatry, University of Bern, Bern, Switzerland; ^2^ Department of Psychiatry, Psychotherapy and Psychosomatics, Psychiatric Hospital, University of Zurich, Zurich, Switzerland

**Keywords:** mental health, women's cycling, sports psychiatry, risk factors, elite athlete, equality

## Abstract

Historically, bicycle riding connoted freedom, independence, and enhanced mental and physical wellbeing for women. Persevering through criticism and moral panic, female cyclists have been competitive since the late 19th century—many earning substantial prize money and prestige. Unfortunately, this progress was not linear in its trajectory and contemporary professional women's cycling continues to be pervaded by structural and cultural challenges, which can have deleterious effects on athlete mental health. Notably, socioeconomic pressures endure, like unstable employment terms, limited team support, and role conflicts. Furthermore, sexual harassment, body shaming, and manipulation may characterize women's experiences with their coaches and teams. Sizable investment gaps between men's and women's teams and competitions often underpin these scenarios of disadvantage. Alongside hindering the development of women's cycling, these adverse circumstances may induce psychosocial risk factors. Within this context, by highlighting sport-specific and sex-specific considerations, the emerging subdiscipline of sports psychiatry can be valuable for protecting and promoting athlete welfare in women's cycling. Raising awareness about extant symptoms, vulnerabilities, contributing behaviours, and systemic issues, can bolster efforts to develop better conditions and care equivalence. To that end, this perspective article draws upon anecdotal and scholarly evidence to provide an overview of psychiatric concerns in women's professional cycling. This informs recommended strategies to improve mental health and advance equality within the sport, which should involve actions from several stakeholders, such as athletes, teams, and governing bodies.

## 1. Introduction

### 1.1. Historical overview of women's competitive cycling

With modernized bicycle designs in the late 1800s, women started riding in greater numbers (1). Gender-based independence was expanding in this epoch, yet female cyclists faced societal judgement, sexism, and pseudoscientific theories (2). Specifically, cycling could remove women from the home and might induce “adverse” physical effects, such as muscle growth or a so-called “strained face” (1). Since bicycling was normatively framed as a masculine pursuit during this time, questions of sexuality and even morality arose (3). Some of this conjecture was conditioned by changes in women's fashion, including billowing skirts, which could impede riding (1). Interestingly, this led women to register for a significant proportion of cycling-specific clothing patents (4, 5). Again, however, clothing innovations were considered both masculine and provocative, and the women who wore them were frequently derided (5).

In the same epoch, women were encouraged to compete in cycling races as an exposition of “speed and skill”; nonetheless, this has been viewed more as a financial ploy exploiting societal curiosity towards women competing in minimal attire, rather than a framework for proto sporting equality (2). Cycling races were considered to be entertainment and therefore, women's ability to attract large crowds to the velodrome conferred value (2). Nevertheless, societal motivations aside, female riders were committed, dedicated, and persistent (6). This was exemplified by early professionals like Tillie Andersen (1875–1965), who worked full-time while simultaneously breaking records and earning sponsorships (6).

Despite this initial popularity, women's professional racing largely diminished until the late 1950s, in part due to the rise of other sports and the availability of automobiles (6). Aside from limited attempts to reestablish women's racing in 1937 and 1958, it was not recognised in the Olympics until 1984, the same year that the female Tour de France was originally instituted (6). Notably, this event has continually experienced disruption and inconsistencies in its scheduling and format.

### 1.2. Women's competitive cycling: contemporary perspectives

Today, women's cycling is overseen by the Union Cycliste Internationale (UCI) and is organized into professional World Teams (WT) and Continental Teams (CT). Races are categorized across four divisions, namely: Women's World Tour, UCI ProSeries, Class 1 and Class 2 (7). As of 2022, there were two hundred and thirty-nine riders over fifteen WT and sixty CT encompassing seven hundred and nine riders (7, 8). For 2023, thirty races were scheduled, comprising both single-day events and stage races (8). There has been an 80% increase in professional riders from 2012 to 2020 (9), and with the introduction of the Paris-Roubaix Femmes in 2021, women's racing is receiving more acclaim (10), as demonstrated by expanding spectator support and more challenging course designs (11, 12).

However, although the notion of a female riding a bike no longer deemed controversial in nearly all societies, the professional sport continues to endure inherent challenges. Many of these have socioeconomic origins, culminating in lower wages and limited resources (13, 14). Other concerns stem from harmful behaviours and unbalanced power dynamics, leading to abusive and coercive experiences (15, 16). While governing bodies seek to strengthen the discipline, there may still be a broader lack of confidence in the sporting ability of professional female cyclists (17), predicated on gender-based dynamics. For instance, Lucas has identified three persistent attitudes: female cyclists lack speed, strength, and stamina; they are not well-suited to long-distance racing; and there is still great sensitivity around public urination (17).

Against this background, substantial socioenvironmental challenges and sport-specific vulnerabilities may be evident across women's cycling, demanding greater scrutiny and tailored responses. Although anecdotal evidence reveals prevailing concerns, the mental health of female riders has been largely neglected in current scientific literature and by other stakeholders in the sport. One professional cyclist articulated this sense of inertia: “There just isn't any other option—so we just have to put up with it” (16). As an emerging subdiscipline, sports psychiatrists have a responsibility to investigate these detrimental paradigms and inform recommendations to safeguard riders. In the authors' opinion, the burgeoning popularity of women's cycling presents an opportunity to facilitate real change. To that end, this perspective paper explores the current mental health landscape within women's cycling, underscoring the necessity of enhanced resources and robust actions to support athlete welfare and advance gender equity.

## 2. Risk factors in women's professional cycling

### 2.1. Socioeconomic determinants

Despite recent progress, substantial economic challenges remain across women's professional cycling, which can present psychosocial burdens for athletes. For instance, the average budget for a men's team may range between ∼20 and 35 million USD depending on category and classification, whereas women's teams may operate within a substantially smaller budget bracket of ∼150,000 USD (14, 18). These disparities can detrimentally affect salaries, access to health resources, and personal self-esteem, amongst other aspects (19).

We acknowledge that laudable developments have occurred in this area, like the introduction of a minimum wage in 2020 (∼35,000 USD annually) (13). Nevertheless, this only applies to WT riders, meaning that 52% of female cyclists must pursue a secondary form of employment and 20% of CT riders receive no remuneration (18). Additionally, many participants, even those earning below the minimum salary, are responsible for expenses, like travel costs or repairs following a crash (20, 21). Consequently, even top-performing athletes still experience economic insecurity, with resulting mental health effects, as exemplified by a two-time Giro Rosa champion working part-time to supplement her income: “it becomes *depressing* (our italics) […] you can only keep putting your whole heart into something for so long when you feel it doesn't matter” (22).

WT men's teams have a mandated minimum wage which is significantly higher than WT women (14, 18, 23). Even still, efforts to equalize pay have been criticized and there is a lack of awareness about sex-based discrepancies [e.g., (24)]. This wage gap correlates with a resource gap, leaving many female athletes without access to the same services as their male counterparts, which may compromise health outcomes (15, 21). For example, men's support staff typically include physical therapy professionals and chef-nutritionists, who assist with recovery (25). Consequently, discussions about the inequities between men's and women's team budgets often illustrate a vicious chain of events; in women's cycling a lack of media coverage leads to a lack of societal interest, in turn leading to a lack of investment. Yet, this neglects the role of the media in perpetuating negative stereotypes about women's sports or choices not to cover them at all; instead, recent viewership figures underline the flawed basis for these decisions and, encouragingly, certain media are beginning to actively champion the sport (21, 26, 27).

Separately, female riders have noted a culture of secrecy around wages and some teams may actively discourage athletes from discussing salaries, concomitantly undermining organizational trust and intra-team relationships (15). Several women have claimed that contracts were misrepresented or changed without review and some found it difficult to receive any remuneration (14, 20). Other riders have described the harmful psychological impact of what they depict as “economic manipulation”, with one conceding: “you quickly reach the point where it just doesn't seem like it's worth it. And there is basically nowhere else to go” (20).

### 2.2. Performance, physiology and sociocultural pressures

Performance, physiological, and sociocultural pressures may engender adverse health concerns amongst some female athletes. As a sport, cycling has historically favored lighter competitors with higher power-to-weight ratios, meaning that unsafe dieting, harmful behaviours, and body shaming can be commonly observed phenomena across both men's and women's disciplines (28). In women's cycling, alarming patterns have emerged, including athletes being forcibly restricted from eating or mocked for their body size by coaches and subsequently developing career-ending eating disorders (ED) (20, 29, 30). One female cyclist outlined a teammate's experiences of “fat-shaming”, which “was so terrible that this poor girl was falling apart by the end of her first training camp” (20). Another revealed how a teammate received a penis-shaped “trophy” from a coach because she was the first rider that he had made cry that season (20).

Additionally, performance-enhancing drugs (PEDs) are becoming an increasingly prominent concern, prompting rigorous testing measures from the UCI (31). Again, external dynamics can contribute to individual behaviours, with some athletes suggesting that support staff encouraged them to change their attitude towards doping (20). Discussing an interaction, one rider stated: “He told me everyone does cortisone, like, that made it okay for me to use it, too?” (20). Significantly, athletes may experience long-term harms from PED use and substantial penalties or suspensions can be enforced when doping infractions are detected (31, 32).

As another controversial issue, attending to basic needs such as urination during competition presents further concerns (15). In certain contexts, sociocultural stigma towards women urinating in public persists, meaning that race facilities must be adapted at additional cost (17). Unlike male riders, the need to cease riding to urinate has been considered a weakness of female cyclists, rather than a biological reality (15). This has been appropriated to justify the reluctance of race organizers to increase stage lengths, as well as being used as evidence to support a lack of competitive drive (17). Yet, the consequences of restricted urination are stark; female cyclists have developed urinary tract infections due to the inability or inconvenience of urinating during competitions, which can require antibiotics and may even lead to severe kidney disease (17). Additionally, riders have been penalised for taking “nature breaks”, inhibiting broader inclinations to urinate (33, 34). Inadequate sanitation facilities are likely to render some women at-risk for intra-race anxiety and other psychological issues since they are unable to manage this basic need (35).

### 2.3. Harmful behaviors, sexual harassment and abuse

Across women's sport, abusive incidents are well-documented, which again raises concern for athlete welfare (36). In cycling-specific settings, commentators have argued this behavior may be used to coerce and manipulate riders into conforming to male-centric training and performance frameworks (37, 38). In this regard, sexist assumptions support the ideology that women are weaker than men and that hostility and aggression can enhance strength and performance (24, 38). Power imbalances within the athlete-coach dynamic may contribute to this, particularly since most coaches in women's cycling are men (37, 39, 40). Wider representations may further encourage humiliation and abasement, as shown by negative media depictions that heighten sexist attitudes towards the sport (41). Moreover, past reports illustrate how cyclists have been dehumanized, verbally and emotionally abused, pitted against teammates, humiliated, and sexually harassed (15, 20, 37, 38). One rider described these situations: “I was nearly held prisoner—on the promise of racing in the World Tour” (20).

Again, a culture of secrecy can predominate within women's cycling teams and beyond, meaning athletes seldom come forward to disclose their experiences, fearing retribution and the reporting protocols (20). Notably, the UCI reporting system is an anonymous email address and the subsequent investigation process has been criticized for lacking full confidentiality (20). This may compromise the mental health of athletes, as again has been identified within the sport: “It was so damaging that it really started to affect my confidence. I began to think that maybe I shouldn't even try to be a cyclist […] and this was when I was already riding for an elite professional women's team” (20).

### 2.4. Organizational structures and rules

Many athletes characterized the structural and organizational aspects of professional cycling as exclusionary and problematic. Dixon and colleagues found that the practice of mixing competitive categories was a participation barrier, with women not being afforded the same opportunities as men (9, 19). Female UCI races are generally shorter and sometimes restructured to have less challenging terrain and/or less climbing, decreasing the prestige of the competition (and prize money) in comparison to men's races (14, 15). For example, the 2023 Men's Tour de France encompassed 21 stages over 3,500 km, as compared to the 2023 Tour des Femmes with 8 stages over 1,029 km (42).

Some race directors continue to have safety concerns about women's abilities, sometimes cancelling or changing races (43). These discrepancies can result in lower self-esteem and possible internalized feelings of being weaker (15). Again, a prominent reason for shorter races assumes that riders are unable to complete the men's course, despite women being physiologically well-suited and even having certain advantages, as shown in endurance sport (44–46). Other controversial issues are evident, including debates about trans athletes competing (47), and questions around physical and mental health management in the wake of tragic accidents (48, 49).

## 3. Evidence of psychopathology in women's cycling

General literature indicates that elite athletes experience mental health concerns with at least the same prevalence rates as the general population, such as for disorders like anxiety, depression, and ED. Across the subpopulation of elite athletes, females may be particularly at-risk for gender-based psychosocial issues, that can lead to the onset of psychopathological disorders or exacerbate extant issues (50). In women's cycling specifically, only a limited number of studies have noted researched psychiatric conditions. However, akin to general elite athlete groups, those that are available have underlined the presence of anxiety, depression, and ED symptoms. For example, in an anonymous survey of *n* = 122 female riders, 13% received treatment for ED and 32% were at-risk for ED (51). Moreover, in this work, 70% of participants reported being pressured to lose weight (51). A sample of *n* = 32 female cyclists provided similar findings, revealing patterns of extreme weight management and weight-related pressures (52), as did qualitative interviews with female riders described by Lichtenstein et al. (53). Work by Kuettel and colleagues suggests that signs of anxiety and depression may be evident in female riders and a narrative analysis of a female professional cyclist's autobiography also highlighted psychosocial pressures and revealed potential indicators of disorder symptomatology (54, 55).

Concerningly, unaddressed mental health issues have disrupted athletes' careers. Per the Cyclists' Alliance survey of *n* = 124 professional female riders, 22% of respondents identified mental health as a reason to consider retiring from the sport (18). Despite not being classified within the International Classification of Diseases as a mental disorder, exercise addiction can also be prominent in women's cycling (56, 57). Exercise addiction may further compromise athlete wellbeing, as it may lead to injury, low energy availability, hypothalamic amenorrhea, or Overtraining Syndrome and associated wide-ranging physical and mental disturbances (58, 59). Equally, depression and anxiety have been found to be correlated with bicycle crashes, and women riders in particular are prone to experience greater psychological challenges following a crash (60). In addition, traumatic brain injury (TBI) has links with depression and preliminary data suggests that there may be gender-based differences in symptomatology post-injury (60, 61).

Though research has examined psychiatric disorders in elite cycling (62), there is a distinct lack of tailored investigations into female athletes and gender-based concerns. This is exemplified by the fact that, of those few studies in this area, the majority use mixed samples [e.g., (63)]. However, with the disparate experiences between female and male riders, it may not be appropriate to derive meaning for both genders equally, as has been outlined in other sporting contexts (64). Further, certain projects that do include both genders may overlook the socioenvironmental realities of women's cycling. One inquiry into the psychological aspects of stress reported that women experience more stress in racing situations versus men, suggesting gender roles expectations as an explanation (65). Another study aiming to understand psychological performance factors in cyclists highlighted how being male was correlated with better endurance performance, based on a group of *n* = 7 women (66). Generally, an insufficient evidence-base around psychiatric symptoms and pharmacological therapeutic interventions amongst female athletes has been identified, as have the difficulties associated with attempting to extrapolate information garnered from male-centric samples (64).

Despite scarce scientific data, there is considerable anecdotal reporting of mental health issues among female cyclists, underlining the need for more academic attention. For example, a group of twelve riders spoke about critical life events on the condition of anonymity for fear of retaliation (20). A former Olympic cyclist, reportedly pressured into silence about an improper team member-coach relationship, died by suicide (67). Furthermore, an Olympic track cyclist also died by suicide, possibly related to TBI (68). Another competitor announced her retirement after enduring experiences with ED (69). Elsewhere, three riders disclosed their experiences of ED, questioning the sporting environment that enables unhealthy behaviors: “Who would put themselves through that?” I wondered. “How could that possibly be OK?” (70–72).

## 4. Recommendations

Based on current anecdotal and scientific evidence then, we believe that there is a need to bolster regulatory programmes, improve awareness, and enhance care provisions in women's cycling. Currently, it is alarming to note that many vulnerable riders may be unwilling or unable to access psychiatric support (73). Elsewhere in sports psychiatry and beyond, researchers have advocated for focus groups, consensus statements, and psychoeducation schemes (74, 75). These may be useful for women's cycling, but alone are not enough; despite incremental progress, historic gender-based disparities still persist across the sport (76, 77). As described, it is also possible that governance structures, reporting protocols, and help-seeking pathways may be inadequate, despite pledges to better support female riders (20, 77, 78). It is likely that there have been previous calls to improve these conditions, which have failed to yield positive results; notably, attempts at establishing an impartial union in service of women cyclists have not been without controversy (79). Within this context, proactive interventions are essential to transform the sport into a safer space and to strengthen its reputation, utilising learnings and evidence from other programs [e.g., (49)]. As the popularity of women's cycling increases, we believe this should be a significant driving force for change.

For optimal success, initiatives must be implemented at-scale and quickly enough to generate positive outcomes. With many risk factors and the adverse conditions of women's cycling, it may be important to distinguish between cycling as a healthy leisure activity and professional riding, which could incur negative physical and mental health outcomes. Some women may enter the sport due to their love of riding and camaraderie or as a pursuit of health but find these qualities lacking in professional contexts. Resultantly, we urge prospective athletes to develop a comprehensive understanding of the potential dynamics of competition and a dedicated self-care plan. Thus, psychoeducational measures in women's cycling may better prepare riders for the realities of participation, which should be disseminated holistically through teams and governing federations. Additionally, recent advancements have been made toward economic equity, but further measures should be enacted to continue progress in this area, owing to the potential psychosocial effects of lower financial security and self-worth (9, 21).

As a basic requirement, women who pursue competitive cycling professionally should be afforded sufficient psychiatric support. Though recommendations around screening, referral, and care have been generally provided by the UCI (80), it remains unclear if this is offered universally, independently, and anonymously, ensuring dignity and respect and protecting against retributive consequences. Despite financial constraints, teams should be mandated to provide these services as an entry criteria for competition, upholding the duty of care towards their riders. Likewise, coaches should undergo thorough evaluation prior to employment in efforts to distinguish their suitability for the role and to mitigate against potentially injurious or abusive practices. Here, governance bodies and teams should be held accountable for delivery, while providing systems for safe, anonymous reporting with the support of athlete unions, and robust disciplinary actions for any harmful behaviours that may occur.

Finally, it is clear that more scientific research is needed in all aspects of women's professional cycling. That being said, in lieu of this, anecdotal evidence from athletes is highly valuable. Though much can be learned from former professionals sharing information (81), we hope that more current athlete-advocates are able to discuss their experiences to improve the welfare of fellow competitors and contemporary conditions within the sport. To that end, regulators, athlete unions, sponsors, and the media can encourage the sharing of these dialogues with the aim of boosting mental health literacy across women's cycling.

## 5. Conclusion

Historically, cycling has held a mercurial place in the lives of women. Though bike riding has contributed to gender-based advancement and has improved mental and physical health, it has also engendered moral panic and denigration. Still, cycling represents an exciting and fulfilling vocation for many, and it is therefore logical that women may pursue a career in the sport.

Yet, due to the structural difficulties and detrimental patterns in current professional contexts at the time of writing, we believe that it is unclear if these benefits are universally attainable. Instead, specific interventions and systemic change are needed to mitigate against gender-based issues and concurrent health disparities. This should involve various stakeholders, such as governing bodies, scientists, teams, and coaches. We also believe that the media have a responsibility to accurately report on these issues and not fulfil either overly positive or negative agendas. Rather than a culture of silence, women's cycling must emphasise a culture of zero tolerance, enforcing measures to safeguard athlete mental health and promote equitable conditions.

## Data Availability

The original contributions presented in the study are included in the article/Supplementary Material, further inquiries can be directed to the corresponding author.
